# Mixed-dimensional InAs nanowire on layered molybdenum disulfide heterostructures *via* selective-area van der Waals epitaxy†

**DOI:** 10.1039/d0na00768d

**Published:** 2021-03-19

**Authors:** Mohadeseh A. Baboli, Alireza Abrand, Robert A. Burke, Anastasiia Fedorenko, Thomas S. Wilhelm, Stephen J. Polly, Madan Dubey, Seth M. Hubbard, Parsian K. Mohseni

**Affiliations:** Microsystems Engineering, Rochester Institute of Technology Rochester NY 14623 USA pkmohseni@rit.edu; NanoPower Research Laboratories, Rochester Institute of Technology Rochester NY 14623 USA; Sensors and Electron Devices Directorate, U.S. Army Research Laboratory Adelphi MD 20783 USA; General Technical Services, LLC Wall NJ 07727 USA

## Abstract

Self-assembly of vertically aligned III–V semiconductor nanowires (NWs) on two-dimensional (2D) van der Waals (vdW) nanomaterials allows for integration of novel mixed-dimensional nanosystems with unique properties for optoelectronic and nanoelectronic device applications. Here, selective-area vdW epitaxy (SA-vdWE) of InAs NWs on isolated 2D molybdenum disulfide (MoS_2_) domains is reported for the first time. The MOCVD growth parameter space (*i.e.*, V/III ratio, growth temperature, and total molar flow rates of metalorganic and hydride precursors) is explored to achieve pattern-free positioning of single NWs on isolated multi-layer MoS_2_ micro-plates with one-to-one NW-to-MoS_2_ domain placement. The introduction of a pre-growth poly-l-lysine surface treatment is highlighted as a necessary step for mitigation of InAs nucleation along the edges of triangular MoS_2_ domains and for NW growth along the interior region of 2D micro-plates. Analysis of NW crystal structures formed under the optimal SA-vdWE condition revealed a disordered combination of wurtzite and zinc-blend phases. A transformation of the NW sidewall faceting structure is observed, resulting from simultaneous radial overgrowth during axial NW synthesis. A common lattice arrangement between axially-grown InAs NW core segments and MoS_2_ domains is described as the epitaxial basis for vertical NW growth. A model is proposed for a common InAs/MoS_2_ sub-lattice structure, consisting of three multiples of the cubic InAs unit cell along the [21̄1̄] direction, commensurately aligned with a 14-fold multiple of the Mo–Mo (or S–S) spacing along the [101̄0] direction of MoS_2_ hexagonal lattice. The SA-vdWE growth mode described here enables controlled hybrid integration of mixed-dimensional III–V-on-2D heterostructures as novel nanosystems for applications in optoelectronics, nanoelectronics, and quantum enabling technologies.

## Abstract 2

Self-assembly of vertically aligned III–V semiconductor nanowires on two-dimensional van der Waals nanomaterials allows for integration of novel mixed-dimensional nanosystems with unique properties for optoelectronic device applications.

## Graphical abstract

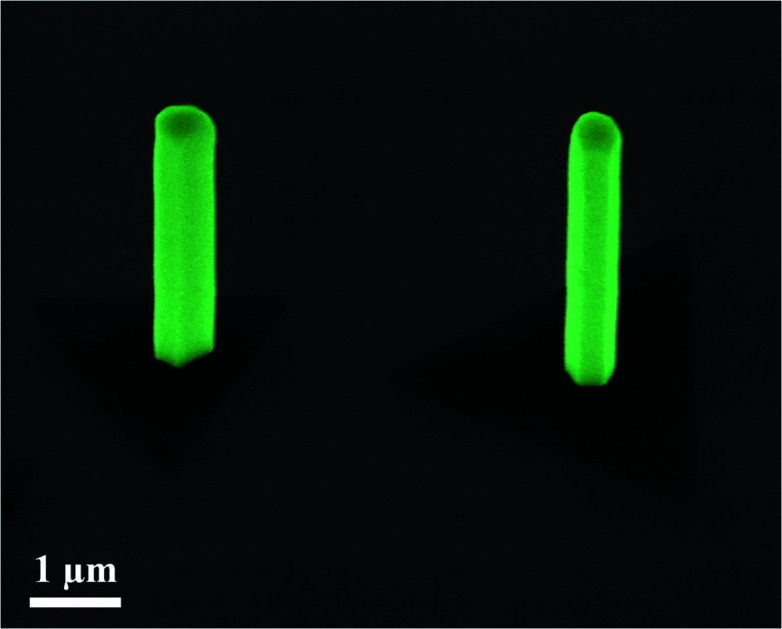

## Introduction

The emergence of two-dimensional (2D) atomically thin materials with novel and tunable physical properties has opened new opportunities for design of next-generation nanoscale electronic devices.^1^ Since the isolation of single layer graphene in 2004,^2^ significant effort has been dedicated to the synthesis and exploration of numerous alternative 2D materials. Among several classes of layered materials such as metal chalcogenides, boron nitride, oxides and oxychlorides, only a few have been successfully isolated in the form of 2D mono- or multi-layers with high crystalline quality. Transition metal dichalcogenides (TMDCs) such as molybdenum disulfide (MoS_2_), molybdenum diselenide (MoSe_2_), and tungsten diselenide (WSe_2_) are among the most extensively studied van der Waals (vdW) layered compounds. Based on the number of transition metal d-electrons, the TMDCs demonstrate metallic,^3,4^ half-metallic magnetism,^5^ semiconducting,^6^ or superconducting characteristics.^7^ For example, semiconductor compounds based on Mo and W, with bandgaps ranging from the visible to the near-infrared, have been widely studied and employed in numerous emerging device applications.^8–10^

In particular, MoS_2_ is one of the most studied 2D TMDC materials owing to its many outstanding properties.^11^ Similar to other TMDC compounds, MoS_2_ exhibits a layered atomic structure with weak vdW interactions between layers and strong intra-layer bonding. Each monolayer of MoS_2_ is a tri-layer sandwich structure, which consists of hexagonal S and Mo atomic layers wherein each Mo atom resides at the center of six S atoms creating a trigonal prism. Interestingly, in its bulk form, MoS_2_ displays an indirect bandgap (*E*_g_ = 1.2 eV), whereas monolayer MoS_2_ has a direct bandgap (*E*_g_ = 1.8 eV).^12^ The transition from bulk MoS_2_ with indirect bandgap to its monolayer form with direct bandgap was predicted theoretically by Li *et al.* in 2007.^13^ In 2010, Splendiani *et al.* investigated this bandgap transition by photoluminescence (PL) of ultrathin MoS_2_ layers and found PL enhancement by decreasing the number of layers. In particular, they observed that monolayer MoS_2_ shows strong emission peaks between 627 nm and 677 nm.^14^ With these properties, MoS_2_ is a great candidate for integration in devices such as field-effect transistors (FETs),^15,16^ light-emitting diodes (LEDs),^17^ photodetectors,^18^ and photovoltaic solar cells.^19^

Heterostructures and superlattices are essential building blocks of electronic and optoelectronic devices. Among the current material integration techniques, chemical epitaxy approaches such as molecular beam epitaxy (MBE) and metalorganic chemical vapor deposition (MOCVD) have offered the highest-quality implementation of more complex heterostructure designs. Chemical epitaxy of two covalently bonded material systems is based on one-to-one chemical bond formation at the heterointerface. For materials with significantly dissimilar lattice structures, misfit dislocations can form at the heterointerface and lead to the formation of extended threading dislocations. This can result in substantial degradation of the properties of the heterostructure. Thus, conventional epitaxy of high crystalline quality heterostructures is generally limited to materials with comparable lattice parameters (*i.e.*, lattice constants, symmetry, thermal expansion coefficient, and polarity).

The class of 2D materials with inert surfaces that are free of dangling bonds provides an alternative pathway for integration of heterostructures *via* van der Waals epitaxy (vdWE), wherein two or more dissimilar 2D materials can be assembled together *via* weak interplanar vdW interactions. Unlike conventional epitaxy, covalent strain sharing is not permitted at the heterointerface during vdWE. Thus, vdW heterostructures can be formed using a wide range of 2D materials with dissimilar crystal structures. For example, high performance photodetectors based on various vdW heterostructures such as MoS_2_/tin diselenide (SnSe_2_),^20^ MoS_2_/graphene/tungsten diselenide (WSe_2_),^21^ and MoS_2_/black phosphorus^22^ have been reported. Moreover, such a hybrid integration technique is not limited to 2D layered materials, and can be applied to materials of dissimilar dimensionality to form mixed-dimensional heterostructures of radically different crystal structures. Recent research on mixed-dimensional vdW heterostructures, as well as their challenges and opportunities, have been reviewed in ref. 23 and 24.

Likewise, semiconductor nanowires (NWs) provide an excellent platform for the formation of complex 3D heterostructures, wherein dissimilar compounds can be monolithically integrated in both radial and axial directions.^25^ This flexibility in design of active nanostructures can provide promising solutions for design of high-performance electronic,^26^ optoelectronic,^25,27^ and photonic^28^ devices. In addition, owning to their large surface area-to-volume ratio and small footprint on the substrate, NWs exhibit excellent strain tolerance. Therefore, III–V NWs offer outstanding potential for integration with a variety of different foreign substrates such as silicon,^29–32^ germanium,^33^ glass,^34^ indium tin oxide,^35^ and 2D vdW surfaces like graphene.^36–41^

In this paper, we present lithography-free hybrid integration of mixed-dimensional heterostructures composed of InAs NWs and MoS_2_ micro-plates *via* selective-area vdWE (SA-vdWE) by MOCVD. The growth parameter space is mapped by altering the V/III ratio, growth temperature, and total flow rate of the precursors. The influence of these parameters on self-assembly of vertically-aligned InAs NWs and growth of parasitic islands on 2D MoS_2_ surfaces is explored. Growth trends are discussed independently for each set of growth trials. The impact of pre-growth surface treatment on MoS_2_ is investigated toward realization of site-controlled SA-vdWE of a single InAs NW on each isolated triangular MoS_2_ domain. The crystal structure of InAs NWs grown under the optimal growth conditions, which are conducive to the formation of a single NW per MoS_2_ micro-plate, is analyzed. Finally, the common sub-lattice registry between vertical InAs NWs and MoS_2_ flakes is discussed using a super-cell model based on the coincident alignment of NW sidewall facets and MoS_2_ micro-island domain edges. The results of this study are expected to provide a foundation for future investigations of mixed-dimensional III–V-on-2D vdW heterostructures for applications in nanoscale optoelectronic and electronic devices.

## Experimental details

The MoS_2_ triangular domains were grown on thermally oxidized (220 nm SiO_2_) Si (100) substrates by atmospheric pressure powder vaporization. Prior to growth, the substrates were cleaned in a piranha solution for 15 min followed by 5 min soaks in deionized (DI) water, acetone, and isopropyl alcohol. Afterwards, 40 μL of perylene-3,4,9,10-tetracarboxylic acid tetrapotassium salt (PTAS) was spin coated on each sample as a seeding layer for growth. Next, ∼20 mg of MoO_3_ powder (Sigma-Aldrich, ≥99.5%) was added to an alumina boat (AdValue Technology), and PTAS-coated substrates were placed on top of the alumina boat face up with a 1.2 mm gap between neighboring samples. The boat was loaded into the center of the furnace. 15–17 mg of sulfur powder (Sigma-Aldrich, 99.8%) was loaded in another alumina boat and placed in a region outside of the furnace where the temperature could be independently controlled by a heating tape. The growth process was performed in the tube furnace at a sample temperature of 700 °C and sulfur temperature of 250 °C. The growth was carried out for 10 min, during which 5 sccm of argon (Airgas, 99.999%) was used as a carrier gas. After the growth was complete, the tube furnace was opened and the argon flow rate was increased to 200 sccm to quench the growth process. The isolated MoS_2_ triangular micro-plates with side lengths ranging between ∼3 μm and 5 μm act as the growth surface in all InAs crystal growth experiments. A top-view optical image of a representative MoS_2_ sample surface used for SA-vdWE experiments is shown in the ESI (Fig. S1).†

For growth of InAs NWs, trimethyl-indium [TMIn; (CH_3_)_3_In] and arsine (AsH_3_) were used as precursors for the supply of group-III and group-V growth species, respectively, in an Aixtron 3 × 2′′ close-coupled showerhead MOCVD reactor. For all growth runs, substrates were heated to the targeted growth temperature under constant AsH_3_ flow. Growth of NWs was initiated by the introduction of TMIn flow into the chamber. After a growth duration of 300 seconds, NW growth was terminated by turning off the TMIn flow. All samples were cooled under a constant AsH_3_ flow.

The optimal SA-vdWE conditions were established by investigating the growth parameter space during three sets of experiments. The three sets of growth runs explored the MOCVD parameter space in the following ranges: (i) V/III ratio was varied between 5 and 250, (ii) TMIn flow rate (*χ*_TMIn_) was varied between 8 and 32 μmol min^−1^ under constant V/III ratio, and (iii) the growth temperature (*T*_G_) was varied between 600 °C and 750 °C, as monitored by susceptor thermocouple setpoint. Based on LayTec EpiTT emissivity-corrected pyrometry measurements, the true temperature at the MoS_2_ surface is ∼120 °C lower than the setpoint temperature. Therefore, thermal decomposition of MoS_2_ domains is not expected under these growth conditions.^42^ For all trials, hydrogen (H_2_) was used as the carrier gas with a total flow rate of 14 L min^−1^, and the reactor pressure was kept constant at 100 mbar. During the initial series of parameter space optimization experiments, no surface treatment was performed on MoS_2_ surfaces prior to loading in the MOCVD reactor. Next, a surface treatment step was performed prior to loading to achieve selective-area single NW synthesis per MoS_2_ domain. This involved dipping MoS_2_ samples in a poly-l-lysine [PLL; (C_6_H_12_N_2_O)_*n*_] solution for a duration of 120 seconds, followed by rinsing in deionized water for 5 seconds.

The morphology of as-grown samples was imaged using a Hitachi S-4000 SEM. The surface roughness of pre-treated and PLL-treated MoS_2_ nano-sheets was measured using a Bruker DI-3000 atomic force microscope (AFM). The crystal structure of NWs was characterized using a FEI F20 high-resolution transmission electron microscope (HR-TEM). TEM lamellae were prepared using a FEI Strata 400 STEM focused ion beam (FIB). Selected-area electron diffraction (SAED) patterns were obtained using the same instrument. Compositional analysis at the InAs/MoS_2_ interface was performed through electron energy loss spectroscopy (EELS) using a Nion UltraSTEM 100 TEM.

## Results and discussions

The main objective of this study is to investigate epitaxy of a covalently bonded III–V compound system upon a 2D vdW layered TMDC film. Here, CVD-grown discrete MoS_2_ micro-plates are used as the growth surface for pseudo-vdWE of InAs NWs. In the first part of this study, the MOCVD growth parameter space is mapped (*i.e.*, V/III ratio, *T*_G_, and *χ*_TMIn_) to find suitable conditions for vdWE of vertical InAs NWs on MoS_2_ micro-plates. To investigate the influence of each parameter independently, one parameter is varied in each set of growth trials while the other two parameters are kept constant. Similar to previously reported studies on integration of III–V NWs with vdW-surfaces, polycrystalline parasitic islands are formed on the growth surface along with vertically-oriented NWs.^38–41^ To evaluate each trial run, tilted-view SEM images of 20 MoS_2_ micro-plates are used to measure the mean NW length and diameter values. In the second part of this study, the objective is to eliminate the formation of parasitic islands and to control crystal growth such that a single NW is formed on each isolated MoS_2_ domain. This is achieved by focusing on the surface and plate-edge characteristics of MoS_2_ micro-plates as well as further tuning of growth conditions.

**Fig. 1:**
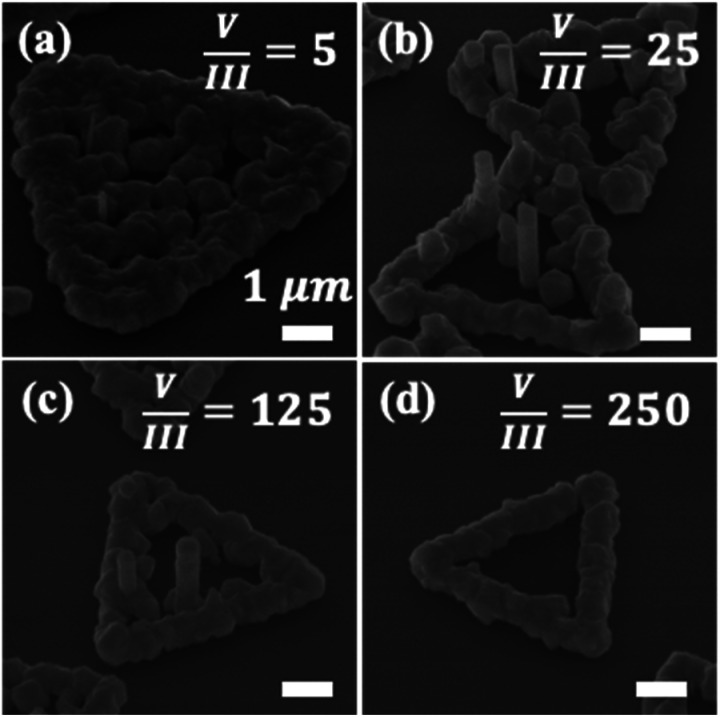
Influence of V/III ratio on the growth of InAs NWs on MoS_2_ micro-plates. 45° tilted-view SEM images of as-grown samples at V/III ratios of (a) 5, (b) 25, (c) 125, and (d) 250, with *χ*_TMIn_ = 16 μmol min^−1^ and *T*_G_ = 650 °C. All scale bars represent 1 μm.

In the first set of experiments, the influence of V/III ratio on the growth of InAs NWs on 2D MoS_2_ micro-plates is investigated. Here, the V/III ratio (*i.e.*, defined by molar flow ratio) is changed over the range of 5 to 250 at a growth temperature of 650 °C. The V/III is modified by altering the molar flow rate of AsH_3_ under a constant molar flow rate of TMIn (*i.e.*, *χ*_TMIn_ = 16 μmol min^−1^). Shown in Fig. 1(a)–(d) are 45° tilted-view SEM images of as-grown samples. At V/III = 5 [Fig. 1(a)], the MoS_2_ micro-plates are fully covered with polycrystalline InAs islands. Increasing the V/III ratio to a range of 25 to 125 results in the formation of NWs along the central region of each MoS_2_ domain, as well as parasitic islands around the plate-edges. At a V/III ratio of 250, InAs crystal formation is strictly limited to the edges of MoS_2_ domains, and no growth takes place along the interior area of the micro-plates.

To understand these results, it should be noted that the sticking coefficient of MoS_2_ micro-plates is not homogenous throughout each domain. When adatoms impinge upon a surface, there are three possibilities: (i) to adsorb on an impingement site, (ii) to migrate on the surface and adsorb on a secondary surface site, or (c) to desorb from the surface. The sticking coefficient is defined as the ratio of the number of adsorbed atoms to the number of atoms that either migrate or desorb.^43^ Here, for instance, the SiO_*x*_ surface that is exposed between neighboring MoS_2_ domains has a very low sticking coefficient, and no crystal growth is observed on the oxide surface. On the MoS_2_ micro-plates, however, the potential for adatom nucleation is greater at the domain edge sites compared to the interior domain surface due to an abundance of available dangling bonds at the plate-edges. Consequently, the domain edges serve as nucleation sites for InAs island growth under all V/III ratios employed in this study. However, the V/III ratio serves to further tune the surface migration of adatoms. Under low V/III ratio growth conditions (*i.e.*, V/III = 5), the growth species can reach the central region of the MoS_2_ microplates, likely due to their enhanced surface migration. A similar effective enhancement of adatom migration under low V/III growth conditions was also observed during InAs NW vdWE on graphene.^38^ In contrast, under high V/III ratio growth conditions (*i.e.*, V/III = 250), crystal growth is limited to parasitic island formation around the edges of MoS_2_ domains, likely due to an effective reduction in adatom surface migration coupled with the availability of bonding sites at the domain edges. At the intermediate V/III ratio range (*i.e.*, V/III = 25 to 125), the growth species that are able to reach the interior segment of the MoS_2_ micro-plates can contribute to the formation of vertical NWs. Therefore, intermediate V/III ratio values represent favorable growth condition for self-assembly of vertical InAs NWs on MoS_2_ domains under the pseudo-vdWE regime. The difference in dimensions of NWs grown under V/III ratios of 25 and 125 is negligible. At V/III = 25, the mean NW length is 1.92 μm and the mean NW diameter is 0.40 μm, whereas at V/III = 125, mean NW length and diameter values of 1.78 μm and 0.44 μm are measured, respectively.

**Fig. 2:**
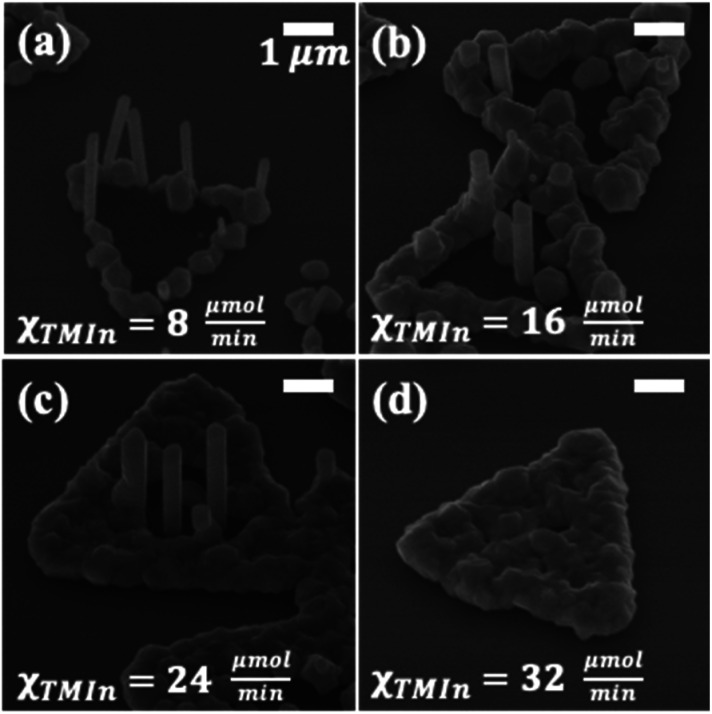
Influence of total precursor flow rate on growth of InAs NWs on MoS_2_ micro-plates. 45° tilted-view SEM images of as-grown samples at *χ*_TMIn_ of (a) 8 μmol min^−1^, (b) 16 μmol min^−1^, (c) 24 μmol min^−1^, and (d) 32 μmol min^−1^, with V/III = 25 and *T*_G_ = 650 °C. All scale bars represent 1 μm.

Next, the influence of growth rate was investigated by altering the total molar flow rate of both metalorganic and hydride precursors (*i.e.*, *χ*_Total_ = *χ*_AsH_3__ + *χ*_TMIn_) under a constant V/III ratio of 25 and growth temperature of 650 °C. To avoid confusion, the results are discussed with references to the molar flow rate of TMIn only (*i.e.*, *χ*_TMIn_). Fig. 2(a)–(d) shows as-grown InAs/MoS_2_ samples formed at various *χ*_TMIn_ values in the range of 8 to 32 μmol min^−1^. At *χ*_TMIn_ = 8 μmol min^−1^, growth is limited to the edges of MoS_2_ micro-plates. This results in the formation of NWs as well as parasitic islands, while no growth is observed along the interior region of the MoS_2_ domains. By increasing the total flow rate of the precursors, the growth front is no longer localized to the MoS_2_ plate-edges, and nucleation proceeds toward the center of each domain. Under the intermediate total flow rate range (*i.e.*, *χ*_TMIn_ = 16 μmol min^−1^ to 24 μmol min^−1^), growth of NWs is predominantly limited to the central region of each MoS_2_ micro-plate, while parasitic islands are observed along the plate-edges. In the case of *χ*_TMIn_ = 32 μmol min^−1^, the MoS_2_ surface is fully coated with a continuous polycrystalline film of InAs, likely formed due to lateral coalescence of parasitic islands.

In the specific case of *χ*_TMIn_ = 8 μmol min^−1^, NWs with mean length and diameter values of ∼2.30 μm and ∼0.26 μm, respectively, are formed around the edge of MoS_2_ micro-plates. Increasing the flow rates to *χ*_TMIn_ = 16 μmol min^−1^ leads to growth of NWs with mean length of ∼1.92 μm and mean diameter of ∼0.40 μm. As the coverage of parasitic islands on MoS_2_ micro-plates increases under high-flow condition of *χ*_TMIn_ = 24 μmol min^−1^, the mean length of NWs reduces to ∼1.64 μm and the mean diameter of NWs increases to ∼0.54 μm. The results that involve varying the total flow rate suggest that by increasing the precursor supply, the growth front moves from the edge of MoS_2_ micro-plates toward the central region (*i.e.*, within ∼2 μm from domain edges). Meanwhile, the aspect ratio of the NWs is reduced due to the role of parasitic islands as the predominant atomic sink.

**Fig. 3:**
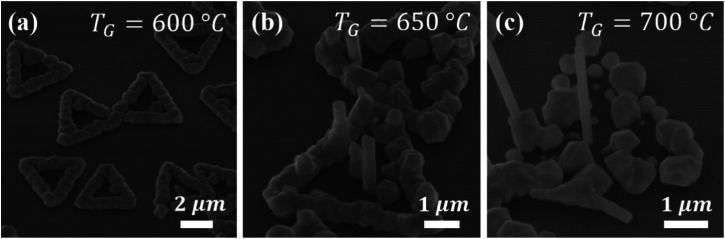
Influence of growth temperature on the synthesis of InAs NWs on MoS_2_ micro-plates. 45° tilted-view SEM images of as-grown samples at *T*_G_ of (a) 600 °C, (b) 650 °C, and (c) 700 °C, with V/III = 25 and *χ*_TMIn_ = 16 μmol min^−1^.

Next, the influence of growth temperature on the formation of InAs NWs on MoS_2_ micro-plates under the pseudo-vdWE regime is investigated. Here, *T*_G_ values of 600 °C, 650 °C, and 700 °C are tested under a constant V/III ratio of 25 and *χ*_TMIn_ = 16 μmol min^−1^. The corresponding growth results are shown in Fig. 3(a)–(c). At *T*_G_ = 600 °C [Fig. 3(a)], the formation of NWs is fully quenched. At elevated growth temperatures of 650 °C and *T*_G_ = 700 °C, however, vertical InAs NWs are self-assembled on MoS_2_ micro-plates, along with parasitic island formation along both central regions and edges of the MoS_2_ domains. For the case of *T*_G_ = 650 °C, the mean NW height and diameter are measured to be 2 μm and 0.35 μm, respectively. By increasing the growth temperature to *T*_G_ = 700 °C, the mean NW length and diameter are increased to 2.35 μm and 0.43 μm, respectively.

In the low temperature range (*i.e.*, *T*_G_ = 650 °C and below), parasitic islands are mainly formed around the edges of MoS_2_ micro-plates and few InAs islands are seen extending toward the interior region. This observation can be understood in terms of temperature-dependent surface mobility of the group-III species and the abundance of dangling bonds at the edges of MoS_2_ domains. The edges of MoS_2_ micro-plates act as favorable nucleation sites for growth species with low surface mobility, where adjacent parasitic islands merge and form a ring-shaped contiguous film. By increasing *T*_G_, and as a result of enhancement in the surface mobility of adatoms, the growth species can migrate toward the interior region of each MoS_2_ domain. It is noted that the density of parasitic islands changes dramatically by further increasing *T*_G_ to 700 °C. This can be attributed to preferential incorporation of diffusive growth species into NW structures instead of parasitic islands.

Growth conditions consisting of V/III = 25, *χ*_TMIn_ = 16 μmol min^−1^, and *T*_G_ = 650 °C permit vdWE of NWs along the interior surface of MoS_2_ domains and simultaneously result in the growth of parasitic islands around the edges of MoS_2_ micro-plates. On the other hand, keeping the V/III ratio and *T*_G_ constant while reducing *χ*_TMIn_ to 8 μmol min^−1^ results in further reduction in the formation of parasitic islands. Moreover, the results of the *T*_G_-dependent growth trials suggest that elevated temperatures are conducive to the formation of NWs and minimum coverage of parasitic islands at the edges of MoS_2_ domains, which is a necessary criterion for the realization of a single NW per MoS_2_ micro-plate. Thus, growth parameters of V/III = 25, *χ*_TMIn_ = 8 μmol min^−1^, and *T*_G_ = 750 °C are selected as the optimal SA-vdWE condition for InAs NWs on MoS_2_ micro-plates.

**Fig. 4:**
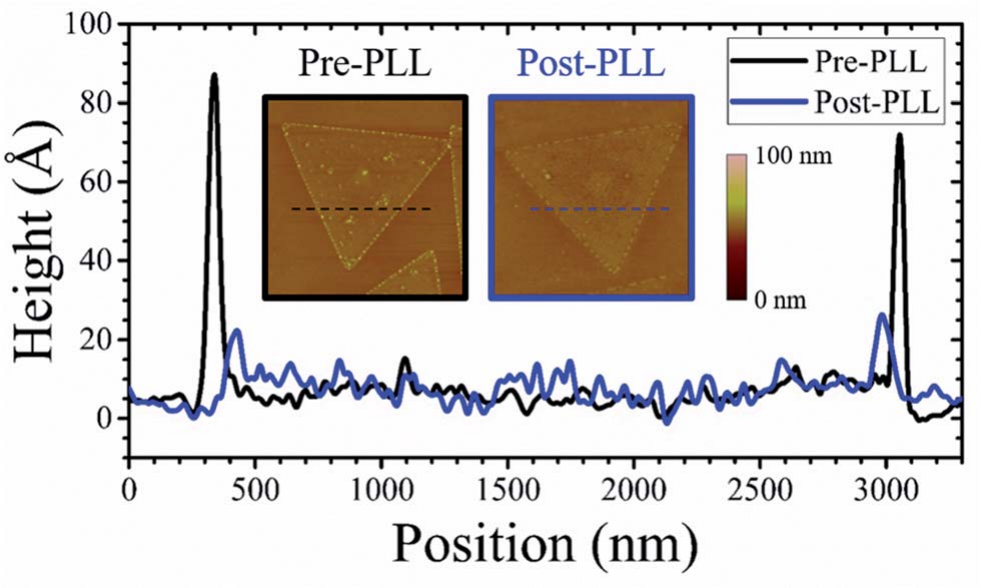
Comparison of AFM height profile of pre- and post-PLL treatment of MoS_2_ micro-plates surfaces. Dashed lines in the AFM image insets indicate the location of height profile measurement.

Next, the influence of a pre-growth PPL surface treatment is investigated. The effect of PLL as a coating reagent for modulating surface charge was reported by Umehara *et al.*^44^ In the current study, PLL is used for charge compensation of dangling bonds at the edges of MoS_2_ micro-plates. Accordingly, the diffusion barrier at the MoS_2_ plate-edges is expected to be affected by passivating the available dangling bonds at those regions. Furthermore, the surface roughness of individual micro-plates is investigated *via* AFM measurements of pre- and post-PLL treated MoS_2_ samples. Fig. 4 shows the height profile of a MoS_2_ micro-plate before and after the PLL treatment, where a pre-PLL treatment height profile is shown in black and a post-PLL treatment height profile is plotted as a blue line. This comparison indicates that the PLL surface treatment reduces the step height at the MoS_2_ plate-edge from ∼90 Å (pre-PLL treatment) to ∼25 Å (post-PLL treatment). This height difference may be attributed to dissolution of MoO_3_ during the surface treatment (*i.e.*, due to PLL treatment and DI water rinse). Representative AFM images of the pre- and post-PLL treatment samples are shown as insets in Fig. 4, highlighted with black and blue borders, respectively. The particles seen on the surface and edges of MoS_2_ micro-plates are significantly reduced after the PLL-treatment. The presence of these particles are reported in various studies and is likely to be unreacted MoO_3_.^16,42^

**Fig. 5:**
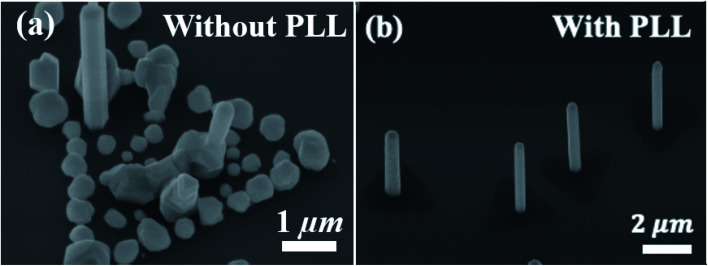
45° tilted-view SEM images of as-grown InAs NWs formed under optimal SA-vdWE growth conditions on MoS_2_ domains (a) without, and (b) with PLL treatment.

The results of optimizing the growth parameter space and the influence of the PLL surface treatment are used for integration of a single InAs NW on individual, isolated, triangular MoS_2_ domains. To this end, samples with and without the PLL treatment are loaded for growth under the previously determined optimal SA-vdWE conditions (*i.e.*, V/III = 25, *χ*_TMIn_ = 8 μmol min^−1^, and *T*_G_ = 750 °C). Tilted-view SEM images of as-grown samples on MoS_2_ micro-plates without and with the PLL treatment are shown in Fig. 5(a) and (b), respectively. As expected, the use of untreated MoS_2_ as the growth surface under the stated MOCVD conditions results in the formation of NWs as well as parasitic islands [Fig. 5(a)]. Since this growth is performed at an elevated temperature, high surface mobility of the group-III species as well as high desorption of the group-V species lead to the formation of discrete parasitic islands, unlike the continuous polycrystalline InAs films formed at lower growth temperatures. However, the same growth conditions, when applied to a PPL treated MoS_2_ sample, enable pristine SA-vdWE of InAs NWs on MoS_2_, such that only one NW grows on each triangular MoS_2_ domain [Fig. 5(b)]. Importantly, parasitic crystallite growth is also eliminated along MoS_2_ plate-edges and surfaces. This approach allows for site-selective self-assembly of free-standing NWs near the central region of each MoS_2_ micro-plate. As noted earlier, PLL likely changes the MoS_2_ surface in two ways: (i) it allows charge compensation of dangling bonds available at the edges of MoS_2_ micro-plates, thereby accommodating an edge-passivation effect and quenching nucleation sites for the formation of parasitic islands; and (ii) it allows for the dissolution of the residual particles and consequently smoothens the MoS_2_ surface, which minimizes the availability of atomic sinks for the nucleation and formation of more than one NW or parasitic islands per MoS_2_ micro-plate. Table 1 summarizes the growth conditions explored in this work and the corresponding influence on the synthesis of InAs NWs on MoS_2_ micro-plates.

**Table 1:** Summary of growth conditions and influence on InAs NW synthesis on MoS_2_

	*χ*_TMIn_ (μmol min^−1^)	*χ*_AsH_3__ (μmol min^−1^)	V/III	*T*_G_ (°C)	PLL	Resulting structures
V/III ratio modulation	16	80	5	650	No	High domain coverage by island growth only
	16	400	25	650	No	Predominant island growth at edges, NW growth toward central regions
	16	2000	125	650	No	Predominant island growth at edges, NW growth toward central regions
	16	4000	250	650	No	Island growth along domain edges only
*χ*_Total_ modulation	8	200	25	650	No	Island and NW growth along domain edges only
	16	400	25	650	No	Predominant island growth at edges, NW growth toward central regions
	24	600	25	650	No	Predominant island growth at edges, NW growth toward central regions
	32	800	25	650	No	Nearly complete domain coverage by island growth only
*T*_G_ modulation	16	400	25	600	No	Island growth along domain edges only
	16	400	25	650	No	Predominant island growth at edges, NW growth toward central regions
	16	400	25	700	No	Partial island growth at edges, NW growth at edges and central regions
Pre-growth treatment	8	200	25	750	No	Partial island growth at edges, NW growth at edges and central regions
	8	200	25	750	Yes	Single InAs NW growth near center of each MoS_2_ domain only

**Fig. 6:**
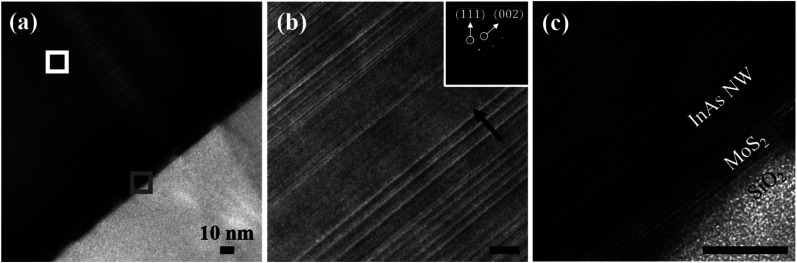
(a–c) HR-TEM images of InAs NW grown under V/III = 25, *χ*_TMIn_ = 8 μmol min^−1^ and *T*_G_ = 750 °C. Panel (b) and (c) show higher magnification views of regions highlighted by white and gray borders in panel (a), respectively. A SAED pattern is shown as an inset in (b). The black arrow represents the NW growth direction.

To investigate the crystal structure of the NWs and to probe the InAs/MoS_2_ interface, TEM lamellae are prepared with a FIB from samples that were grown under the optimized SA-vdWE condition after PLL-treatment. Fig. 6 shows HR-TEM images of a NW acquired along the 〈011̄〉_ZB_ zone axis of the cubic phase. The base of the NW is shown in Fig. 6(a), and the NW growth direction along 〈1̄1̄1̄〉_ZB_ is indicated with a black arrow in Fig. 6(b). The approximate locations of the HR-TEM images shown in Fig. 6(b) and (c) is illustrated in Fig. 6(a) using solid white and gray borders, respectively. Firstly, it is noted that under the SA-vdWE conditions (*i.e.*, V/III = 25, *χ*_TMIn_ = 8 μmol min^−1^ and *T*_G_ = 750 °C), the crystal structure throughout the entire length of the NW consists of a combination of zinc-blend (ZB) and wurtzite (WZ) phases. Such a characteristic disordered crystal structure with a high density of stacking faults and rotational twin planes^45^ was also observed for InAs NWs grown on graphene under the vdWE mode,^37,41^ as well as for InAs NWs grown *via* the SAE mode on Si substrates.^46–48^ The SAED pattern, shown as an inset in Fig. 6(b), exhibits streaking along the 〈1̄1̄1̄〉_ZB_ axis, which confirms the disordered crystal phase of the InAs NW. Next, the interface of the InAs NW and MoS_2_ micro-plate is shown in a high-magnification micrograph [Fig. 6(c)]. Here, at the location of the NW growth, a total of five vdW-bonded MoS_2_ layers are observed in the micro-plate. Despite the large NW diameter (*i.e.*, >350 nm), misfit dislocations are not found at the InAs/MoS_2_ interface, and the NW crystal is free of threading dislocations. This is likely due to the absence of strain sharing between the dissimilar lattices. In Fig. 6(c), an abrupt heterointerface is observed that is absent of compositional grading or transitional phases. The average interplanar separation in the axial direction is measured to be ∼3.518 Å and ∼6.125 Å along the InAs and MoS_2_ phases, respectively. Compositional analysis of the InAs/MoS_2_ interface performed using EELS is detailed in Fig. S2 of the ESI† document.

**Fig. 7:**
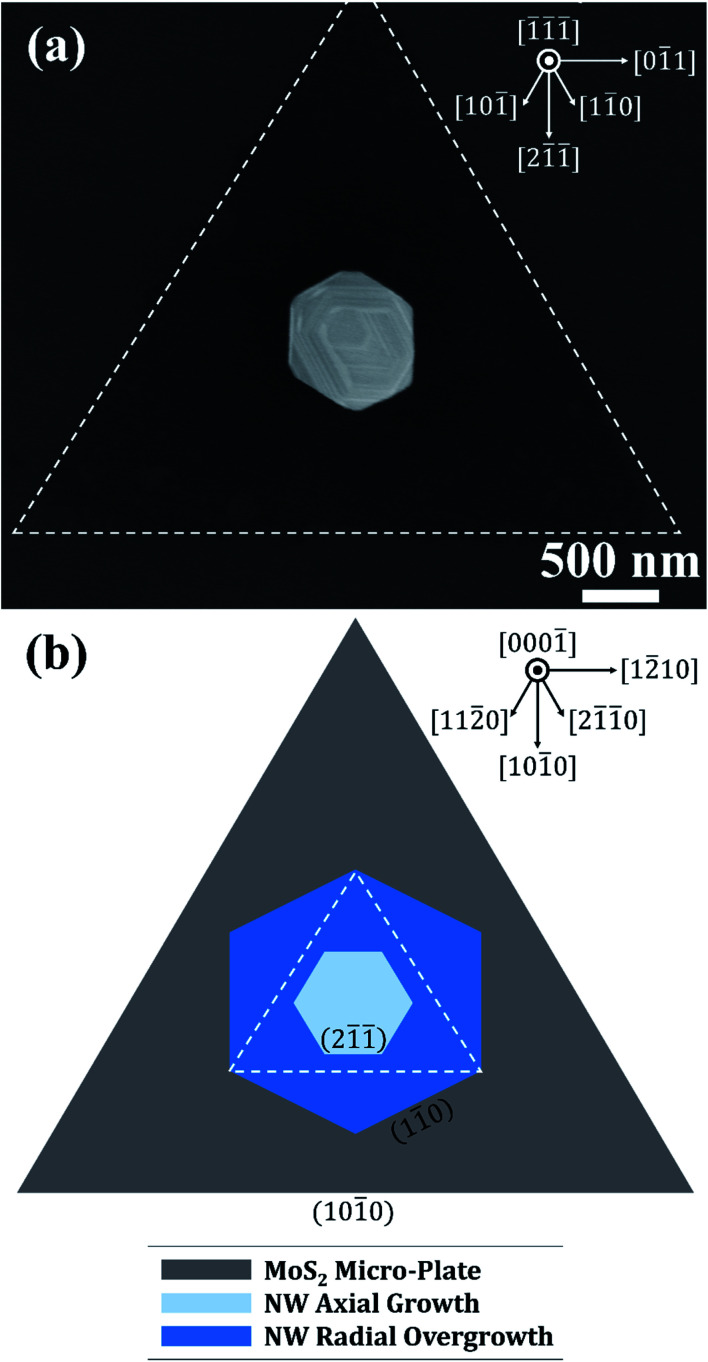
(a) Top-view SEM image of a single InAs NW on a triangular MoS_2_ domain with micro-plate edges indicated by a dashed white border. (b) Schematic representation of the sidewall faceting structure observed in (a), highlighting the in-plane orientation relationships between the NW core segment (light blue), radially overgrown NW shell segment (dark blue), and triangular MoS_2_ domain. Cubic and hexagonal lattice directions are indicated by compasses at the top-right corners of (a) and (b), respectively.

The lattice alignment and in-plane orientation of InAs NWs relative to the triangular MoS_2_ micro-plates are considered next. Fig. 7(a) shows a plan-view SEM image of a representative InAs NW grown under the optimized SA-vdWE conditions on a triangular MoS_2_ micro-plate, where the sides of the latter are outlined by white dashed lines. The hexagonally cross-sectioned NW is situated near the center of the MoS_2_ domain. From the plan-view SEM image, two separate sets of NW sidewall facets (*i.e.*, two families of sidewall planes) can be observed. The sidewall planes can be identified with reference to SAED patterns obtained from NWs lifted out of the as-grown sample using FIB. The interior facets of the NW are principally {21̄1̄}-oriented, and three of six visible interior facets are aligned in a parallel orientation relative to the three known {101̄0}-oriented sides of the MoS_2_ micro-plate.^49^ The exterior NW sidewall facets, however, appear to be rotated 30° relative to the interior facets. Therefore, the exterior facets of the NW are principally {11̄0}-oriented. The legend at the top-right corner of Fig. 7(a) indicates the corresponding directions along the cubic InAs lattice. The apparent rotation of the planar sidewall structure can likely be attributed to the evolution of radially-overgrown NW shell layers upon an axially-grown NW core segment. This stems from preferential adatom nucleation along NW sidewalls, initiated at the low-energy vertices of the core segment sidewalls,^50^ primarily due to the relative lack of nucleation sites on the vdW surface. Preferential nucleation of adatoms on NW sidewalls leading to modulation of the faceting structure from the {21̄1̄}-orientation (*i.e.*, central core segment) to the {11̄0}-orientation (*i.e.*, external shell segment) has been previously reported for III–V NWs synthesized under different growth modes.^51–55^

A plan-view diagrammatical representation of the in-plane orientation of the axially-grown NW core segment (light blue, interior hexagon) and radially-overgrown NW shell segment (dark blue, exterior hexagon) relative to the triangular MoS_2_ micro-flake (gray triangle) is shown in Fig. 7(b). The legend at the top-right corner of Fig. 7(b) indicates crystallographic directions corresponding to the hexagonal MoS_2_ lattice. The representative planar indices of the interior and exterior NW sidewall facets and the exterior MoS_2_ plate-edge facets are labelled. As a guide to the eye, the white dashed triangular border depicts the coincident sidewall orientations of the NW core segment and the MoS_2_ micro-flake. Since the NW core segment with {21̄1̄}-orientated sidewall facets is formed directly on the MoS_2_ surface, the epitaxial relationship between these two components, which enables vertically-oriented NW growth during SA-vdWE, can be understood in terms of the coincident in-plane alignment of their lattices.

**Fig. 8:**
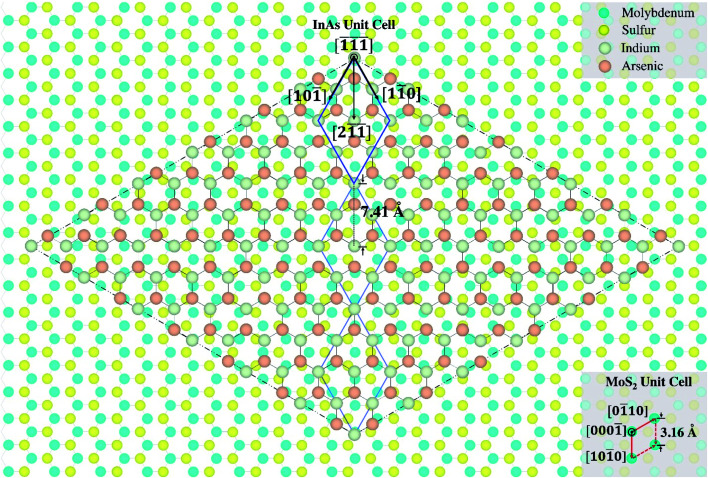
Plan-view model of a nearly-commensurate super-cell configuration composed of a (1̄1̄1̄)-oriented InAs cubic lattice on (0001̄)-oriented MoS_2_ hexagonal lattice, consistent with the alignment of the NW core segment relative to the MoS_2_ domain shown in Fig. 7. The 2 × 2 InAs unit cell and MoS_2_ unit cell are highlighted using blue and red borders, respectively. The legend shows Mo, S, In, and As atoms in blue, yellow, gray, and orange, respectively. A pseudo-commensurate relationship is indicated by 3 multiples of the InAs unit cell in the [21̄1̄] direction and coincident positions of In and Mo atoms at the corners of the InAs sub-cell, which is shown as the overlaying rhombus-shaped lattice.

Based on the observed symmetrical orientation and coherent alignment between the NW and 2D micro-plate, a model for a nearly-commensurate super-cell lattice configuration for 〈111〉-oriented InAs NWs on MoS_2_ is proposed. Fig. 8 depicts the relative atomic arrangement of InAs and MoS_2_ compounds on equivalent (1̄1̄1̄)- and (0001̄)-oriented surfaces, where Mo-, S-, In-, and As-atoms are shown in blue, yellow, gray, and orange, respectively. The cubic 2 × 2 InAs unit cell is shown as a reference (highlighted by blue borders), along with the primitive unit cell of hexagonal MoS_2_ (highlighted by red borders). As indicated in Fig. 8, a common sub-lattice is formed such that a distance equal to three multiples of the cubic InAs unit cell along the [21̄1̄] direction is nearly commensurate with a 14-fold multiple of the Mo–Mo (or S–S) spacing along the [101̄0] direction of the hexagonal lattice. The proposed lattice registry is in agreement with observations based on the top-view SEM image shown in Fig. 7, where the sidewall facets of the InAs core segment are parallel to the MoS_2_ micro-flake facets.

## Conclusions

In summary, SA-vdWE of vertically aligned InAs NWs on isolated MoS_2_ domains is demonstrated *via* MOCVD. The growth parameter space is explored in order to optimize positioning of single InAs NWs on discrete MoS_2_ micro-plates with one-to-one NW-to-MoS_2_ placement. The influence of pre-growth surface treatment is examined using PLL. The SA-vdWE growth condition is achieved using a combination of V/III = 25, *T*_G_ = 750 °C, and *χ*_TMIn_ = 8 μmol min^−1^ on PLL-treated MoS_2_ micro-plates. The NWs grown under these conditions exhibit a disordered crystal lattice, similar to the case of vdWE of InAs on graphitic surfaces. Sidewall facet modulation resulting from radial overgrowth on axially-grown NW core segments is observed. The sidewall surfaces of the NW core segment are shown to have a coincident alignment with the MoS_2_ plate-edges. A model for a nearly-commensurate atomic arrangement of cubic InAs on the hexagonal MoS_2_ lattice is presented, wherein a three-fold multiple of the cubic InAs unit cell along the [21̄1̄] direction coincides with a 14-fold multiple of the Mo–Mo (or S–S) spacing along the [101̄0] direction of the MoS_2_ hexagonal lattice, thereby forming a common sub-lattice. Similar trends are expected for SA-vdWE growth of alternative combinations of III–V compound semiconductor nanostructures and TMDC monolayer materials. The SA-vdWE growth mode enables the synthesis of a new class of mixed-dimensional hybrid nanosystems for heterojunction optoelectronics device applications and quantum communications enabling technologies.

## Conflicts of interest

There are no conflicts to declare.

## Supplementary Material

NA-003-D0NA00768D-s001
