# Supplementary material for: Mixed-dimensional InAs nanowire on layered molybdenum disulfide heterostructures via selective-area van der Waals epitaxy
Source: Nanoscale Adv. 2021 Mar 19;3(10):2802–11. doi: 10.1039/d0na00768d (PMC9419183; doi:10.1039/d0na00768d)
Supplement: NA-003-D0NA00768D-s001 [file NA-003-D0NA00768D-s001.pdf]

Electronic Supplementary Material (ESI) for Nanoscale Advances.
This journal is © The Royal Society of Chemistry 2021

– Supporting Information –

Mixed-dimensional InAs Nanowire on Layered Molybdenum Disulfide Heterostructures via Selective-Area van der Waals Epitaxy

Mohadeseh A. Baboli[1,2], Alireza Abrand[1,2], Robert A. Burke[3,4], Anastasiia Fedorenko[1,2], Thomas S. Wilhelm[1,2], Stephen J. Polly[2], Madan Dubey[3], Seth M. Hubbard[1,2], and Parsian K. Mohseni[1,2,*]

[1]*Microsystems Engineering, Rochester Institute of Technology, Rochester, NY 14623, USA*

[2]*NanoPower Research Laboratories, Rochester Institute of Technology, Rochester, NY 14623, USA*

[3]*Sensors and Electron Devices Directorate, U.S. Army Research Laboratory, Adelphi, MD 20783, USA*

[4]*General Technical Services, LLC, Wall, NJ 07727, USA*

**Author to whom correspondences should be addressed. E-mail: pkmohseni@rit.edu*

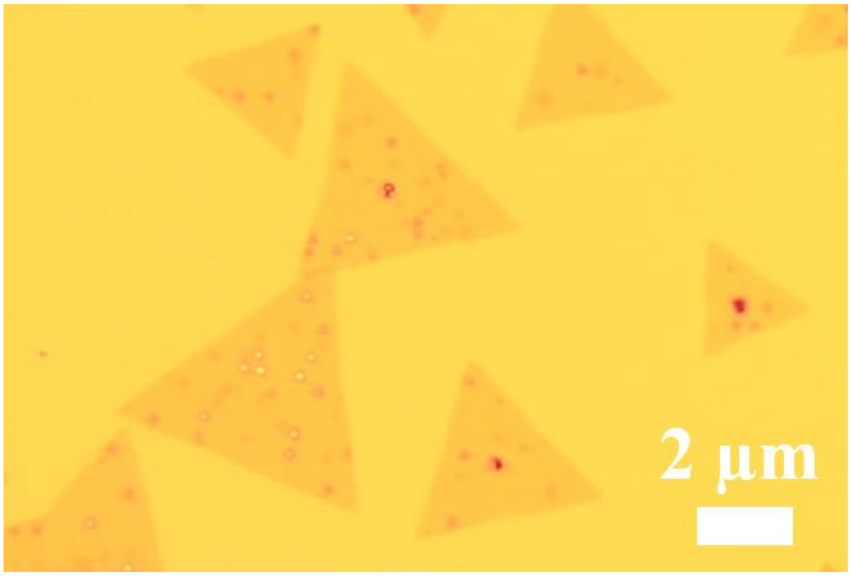

Figure S1. Optical image of MoS_2 micro-plates.

Figure S1 shows top-view optical image of isolated MoS_2 micro-plates. The particles on the surface of these MoS_2 domains are likely to be MoO_3 residues and multilayered MoS_2 sub-domains.

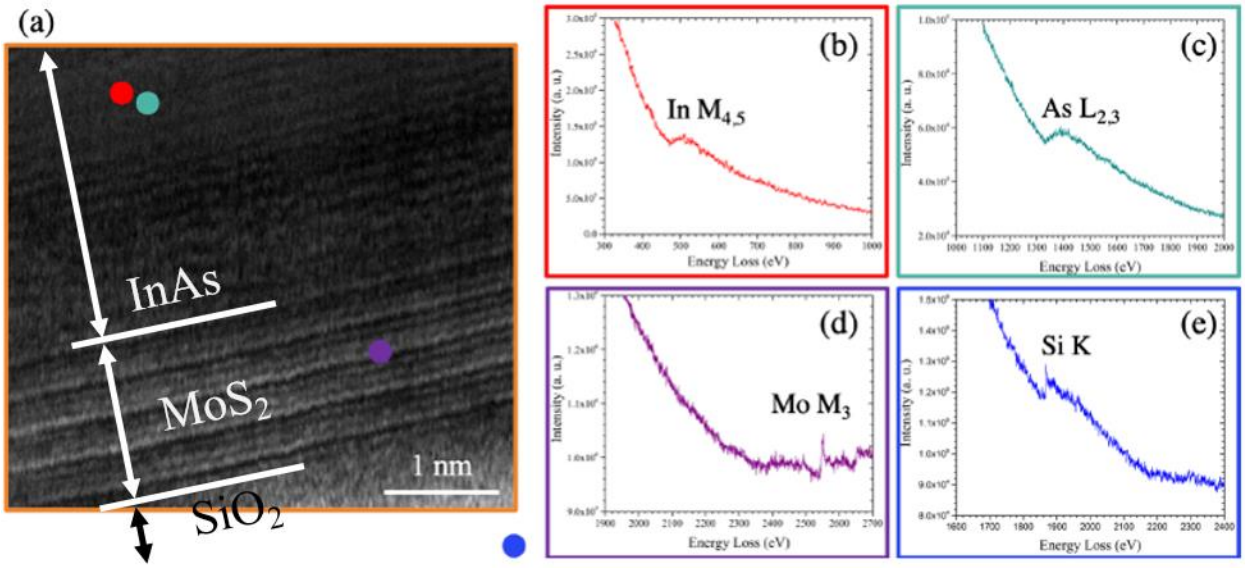

Figure S2. (a) HR-TEM image obtained from the InAs/MoS_2 interface. Electron energy-loss spectra (EELS) showing ionization edges corresponding to (b) In, (c) As, (d) Mo, and (e) Si elements. The color coded borders correspond to the matching markers in the HR-TEM image in (a). The Si EELS data is collected from the underlying Si substrate, which resides outsides the viewing field of (a).

Compositional analysis of the InAs/MoS_2 interface was carried out using electron energy-loss spectroscopy (EELS) in a NION ultra-STEM 100 instrument. Figure S2(a) shows the HR-TEM image of multilayer MoS_2 and the base of an InAs NW. The EELS spectra with corresponding peaks marked for In $M_{4,5}$, As $L_{2.3}$, Mo M_3, and Si K ionization edges are shown in Figure S2(b), (c), (d), and (e), respectively. The locations where the EELS data was collected are shown on Figure S2(a) with red, green, violet, and blue dots representing elemental In, As, Mo, and Si, respectively. Here, five layers of MoS_2 can be counted. During CVD synthesis of MoS_2 domains on SiO_2/Si, high concentrations of MoO_3 and S vapor precursors initiates a self-seeding nucleation mechanism, resulting in the formation of a central particle-type feature with a multilayer structure. In contrast, by lowering the concentration of reactants in the CVD chamber, the 2-D planar nucleation mechanism leads to production of monolayer or bilayer MoS_2 [1].

Reference

[1] D. Zhou, H. Shu, C. Hu, L. Jiang, P. Liang, and X. Chen, *Cryst. Growth Des.*, 2018,**18**, 1012–1019.
